# Investigation on the Microbial Diversity of Fresh-Cut Lettuce during Processing and Storage Using High Throughput Sequencing and Their Relationship with Quality

**DOI:** 10.3390/foods11121683

**Published:** 2022-06-08

**Authors:** Yeting Sun, Xiaoyan Zhao, Yue Ma, Zhihong Ma, Zhaoying He, Wenting Zhao, Pan Wang, Shuang Zhao, Dan Wang

**Affiliations:** 1 Institute of Agri-food Processing and Nutrition, Beijing Key Laboratory of Agricultural Products of Fruits and Vegetables Preservation and Processing, Key Laboratory of Vegetable Postharvest Processing, Ministry of Agriculture and Rural Affairs, Beijing Academy of Agriculture and Forestry Sciences, Beijing 100097, China; syt_nercv@163.com (Y.S.); xiaoyanzhao001@163.com (X.Z.); mayue@iapn.org.cn (Y.M.); zhaowenting@iapn.org.cn (W.Z.); wangpan@iapn.org.cn (P.W.); zhaoshuang@iapn.org.cn (S.Z.); 2 Institute of Quality Standard and Testing Technology, Beijing Academy of Agriculture and Forestry Sciences, Beijing 100097, China; mazh@brcast.org.cn (Z.M.); hzy@brcast.org.cn (Z.H.)

**Keywords:** fresh-cut lettuce, processing and storage, microbial diversity, sensory quality

## Abstract

Microbial community distribution in vegetables can affect their quality. This study analyzed the distribution of the microbial community at various stages during processing and storage with the microbial diversity analysis, and evaluated the correlation between the dominant bacteria and sensory quality of lettuce using correspondence analysis with multiple regression analysis. Results showed that the process of washing, cutting, then disinfection and dewatering could change the community distribution and dominant bacteria in lettuce, and maintain better texture, morphology, aroma, color qualities of lettuce. The total number of colonies and relative abundance of *Xanthomonas* in fresh-cut lettuce decreased, while *Afipia* and *Ralstonia* increased during processing and pre-storage (storage for 6 h, 12 h and 1 d). After storage for 3 d, the total number of colonies in lettuce increased (more than 5 log CFU/g), especially the relative abundance of *Pseudomonas*, which led to the obvious deterioration of the sensory quality of lettuce. Throughout the process, the number of *Bacillus cereus*, *Staphylococcus aureus*, and *E. coli* was less than 100 CFU/g and 3 MPN/g. The number of typical pathogenic bacteria, *Salmonella*, *Listeria monocytogenes* and *E. coli* O157:H7, was below the detection limit. Overall, the prevention and control of psychrotrophic *Pseudomonas* in lettuce was still necessary. These results will provide useful information for the fresh-cut lettuce industry.

## 1. Introduction

Fresh-cut lettuce is a leafy vegetable that is widely consumed in the world. It is commonly eaten raw because of its refreshing taste and high nutritional value; therefore, the safety of fresh-cut lettuce has attracted great attentions from people [1]. The process of fresh-cut lettuce includes: removing the damage, cleaning, cutting, disinfection, dewatering, packaging and storing; every step in the processing may affect the microbial community distribution in lettuce [2,3]. Research has shown that the steps of removing the damage and cleaning can reduce the microorganisms on the surface of fresh-cut lettuce, and can also change their community distribution. Holvoet et al. found that the washing step would disperse or introduce microorganisms on fresh-cut produce [4]. Frimpong et al. found that the cutting step may increase the proportion of *Bacillus cereus* and *Cronobacter sakazakii* in lettuce [5]. This may be because the pressure difference between the vegetables and the water produced by fresh-cut lettuce under low temperature processing conditions may allow the pathogens in the water to internalize into the tissues of the vegetable [6]. Studies have shown that disinfection, dewatering, packaging and low-temperature treatment can reduce the growth and proliferation of microorganisms and improve the microbial safety of lettuce [7,8,9]. However, the correlation between the distribution and change of the microbiota and the quality of fresh-cut lettuce at various stages during processing and storage has not been reported.

Microbial contamination is one of the main factors leading to the decline in quality of fresh-cut lettuce after processing; moreover, the microbial diversity of fresh-cut lettuce may cause inconsistent quality in the lettuce [10]. For example, the lipopeptide produced by *Pseudomonas* strains can promote cell-wall-degrading enzymes to the plant surface, leading to the spoilage of leafy vegetables [11]. Spoilage microorganisms secrete lytic enzymes, which cleave the linkages of the plant cellulose–hemicellulose network and pectin, thereby weakening the plant cell wall. Lipase is used to degrade lipid constituents of plants [12,13]. Plant proteins and proteinaceous materials are degraded into polypeptides and amino acids, and starch is broken down into maltose by amylase, which eventually lead to spoilage of vegetables [14]. However, the dominant populations for contamination on lettuce are not known and clarifying which microorganisms infect fresh-cut lettuce at what stage during processing and storage, contributing to the quality decline, will provide useful information for the development of control methods.

Traditional culture approaches are difficult to isolate due to unculturable microorganisms meaning the results do not reflect complete bacterial information. Culture-independent high-throughput sequencing (HTS) technology is used to analyze the microbial diversity in food and provide information about the microbial community structures. This methodology unveiled the microbial community diversity and dominant genera in industrial Zhacai paocai and disinfected lettuce [15,16].

To our best knowledge, there is no report on whether processing and cold storage can positively regulate the community distribution in fresh-cut lettuce and affect the sensory quality of fresh-cut lettuce. The purpose of this study was to understand the dynamics of complex microbial communities, and to identify the corresponding dominant bacteria that may lead to improved hygiene strategies to reduce the risk of microbial contamination during fresh-cut lettuce processing and cold storage. Revealing the relationship between the dominant genera and the decline in the quality of lettuce will make a greater contribution to the safety control of the final processing of lettuce.

## 2. Materials and Methods

### 2.1. Lettuce Samples and Processing

Green leaf lettuces (*Lactuca sativa* Var. *Crispa* L.) were obtained from Guoxiangsiyi market (Zhanghua Road, Beijing, China) on the day of the experiment and put into a sterile sampling bag (20 × 32 cm in size). Then, they were immediately transported to the laboratory, retained and marked as fresh (F). Lettuces were stored in a cold room at 4 °C for precooling, followed by processing. Lettuces had their outer leaves removed and the inner leaves were rinsed under distilled water for 30 s to remove the soil (W). Stems of the lettuce were cut off and the remaining lettuces were then cut into two halves with a kitchen knife. Lettuces were then disinfected with 100 ppm and 50 ppm sodium hypochlorite (adjusted to pH 6.5 with citric acid) for 2 min, respectively. The lettuces were rinsed again with distilled water and the obtained samples were dewatered using an automatic spinner. Then, they were retained and marked as cut, disinfected and dewatered (CD). Finally, lettuces were placed into polyethylene plastic bags (42.8 cm × 29.1 cm × 4.25 μm) and sealed. The parameters of the polyethylene plastic bag were that they had an oxygen transmission rate of 1113.66 cm^3^/m^2^ 24 h 0.1 MPa and a carbon dioxide transmission rate of 3669.42 cm^3^/m^2^ 24 h 0.1 MPa. Samples weighed approximately 200 g per bag. Samples were stored at 4 °C for 8 days, then samples of 6 h, 12 h, 1 d, 2 d, 3 d, 4 d, 6 d and 8 d were used for subsequent sensory and microbiological diversity analyses, as well as colony counts (retained and marked as s6h, s12h, s1d, s2d, s3d, s4d, s6d and s8d, respectively).

### 2.2. DNA Isolation and Illumina MiSeq Sequencing Analysis

Illumina MiSeq sequencing analyses were detected by Majorbio Bio-Pharm Technology Co., Ltd. (Shanghai, China). Each lettuce sample was mixed and divided into four even parts, samples were frozen into powder with liquid nitrogen and stored in a −80 °C refrigerator (902-ULTS, Thermo Electron Co., Waltham, MA, USA). The powder (1 g) was weighed and the total genomic DNA was extracted using the FastDNA SPIN Kit for soil (MP Biomedicals, Solon, OH, USA) according to the manufacturer’s instructions. The DNA extract was checked on 1% agarose gel, and DNA concentration and purity were performed using a NanoDrop 2000 UV-vis spectrophotometer (Thermo Scientific, Wilmington, NC, USA). The bacterial 16S rRNA genes were amplified with primer pairs 338 F (5′-ACTCCTACGGAGGCAGCAG-3′) and 806 R (5′-GGACTACHVGGGTWTCTAAT-3′) by an ABI GeneAmp^®^ 9700 PCR thermocycler (ABI, Waltham, MA, USA). The expected size of the amplicon is 750 bp. The PCR amplification of 16S rRNA gene was performed as follows: initial denaturation at 95 °C for 3 min, followed by 35 cycles of denaturing at 95 °C for 30 s, annealing at 55 °C for 30 s and extension at 72 °C for 45 s, single extension at 72 °C for 10 min, and end at 10 °C. The PCR mixtures contained 5× FastPfu buffer 4 μL, 2.5 mM dNTPs 2 μL, forward primer (5 μM) 0.8 μL, reverse primer (5 μM) 0.8 μL, FastPfu polymerase 0.4 μL, BSA 0.2 μL, template DNA 10 ng and, finally, ddH_2_O up to 20 μL. PCR reactions were performed in triplicate. The PCR product was extracted from 2% agarose gel and purified using the AxyPrep DNA Gel Extraction Kit (Axygen Biosciences, Union City, CA, USA) according to the manufacturer’s instructions, and quantified using Quantus^TM^ Fluorometer (Promega, San Diego, CA, USA). Purified amplicons were pooled in equimolar amounts and paired-end sequenced on an Illumina MiSeq PE300 platform/NovaSeq PE250 platform (Illumina, San Diego, CA, USA) according to the standard protocols by Majorbio Bio-Pharm Technology Co., Ltd. (Shanghai, China); the depth of each sample was at least 30,000 sequences. 

### 2.3. Processing of Sequencing Data

The raw 16S rRNA gene sequencing reads were demultiplexed, quality-filtered by fastp version 0.20.0 [17] and merged by FLASH version 1.2.7 [18] with the following criteria: (i) the 300 bp reads were truncated at any site receiving an average quality score of <20 over a 50 bp sliding window, the truncated reads shorter than 50 bp were discarded, and reads containing ambiguous characters were also discarded; (ii) only overlapping sequences longer than 10 bp were assembled according to their overlapped sequence. The maximum mismatch ratio of the overlap region is 0.2. Reads that could not be assembled were discarded; (iii) Samples were distinguished according to the barcode and primers, and the sequence direction was adjusted, requiring exact barcode matching and a 2 nucleotide mismatch in primer matching.

Operational taxonomic units (OTUs) with a 97% similarity cutoff were clustered using UPARSE version 7.1 [19,20], and chimeric sequences were identified and removed. The taxonomy of each OTU representative sequence was analyzed by RDP Classifier version 2.2 [21] against the 16S rRNA database (e.g., Silva v138) using a confidence threshold of 0.7. The microorganism community composition of the samples was mainly based on the results of the OTU, the community structure was classified to the genus level, and the dominant species at the genus level were compared to reflect the differences in species richness.

### 2.4. Microbiological Analysis

Lettuces (25 g) were put into a sterile bag (S05D, Land Bridge Technology Co., Ltd., Beijing, China) and mixed with 225 mL PBS using a beating homogenizer (BagMixer 400 W, Interscience Lab Inc., Lyon, France) for 5 min. The homogenized solution (1 mL) was serially diluted at a ratio of 1:10 with PBS. Next, 0.1 mL of the diluted suspensions was spread flat on plant count agar and incubated at 37 °C for 48 h for the total number of colonies [22]. MYP (mannitol–egg yolk–polymyxine) agar was used to count *Bacillus cereus* colonies and inoculated at 35 °C for 24 h [23]. Xylose lysine deoxycholate agar (Difco) and Baird-Parker agar (Difco) were inoculated at 35 °C for 24 h for the enumeration of *Salmonella* and *Staphylococcus aureus*, respectively [24]. Palcam agar was inoculated at 30 °C for 48 h for the enumeration of *Listeria monocytogenes* [25]. MacConkey agar supplemented with 2.5 mg/L potassium tellurite solution and 0.05 mg/L cefixime (Land Bridge Technology Co., Ltd., Beijing, China) and was incubated at 37 °C for 24 h for *E. coli* O157:H7 colony counting [26]. Eosin methylene blue agar (EMBA) was incubated at 37 °C for 24 h for *E. coli* colony counting [27]. Sample dilutions were serially diluted in ten-fold increments using a peptone diluent (0.1%). Samples were analyzed to detect the most probable number (MPN) of bacteria according to the method in the bacteriological analytical manual [28].

### 2.5. Sensory Analysis

A panel of 15 sensory reviewers evaluated the sensory characteristics (appearance, color, texture and smell) of lettuces using the method of Park et al. with slight modifications [29]. Before performing sensory analysis, lettuces were put into sterile plastic boxes for sensory analysis with different treatments. All sensory evaluations were carried out in a room, with a total of 15 independent evaluation rooms. The evaluators were required to evaluate the sensory characteristics of the lettuces as per the following criteria (Table 1):

### 2.6. Statistical Analyses

Data were statistically analyzed with one-way analysis of variance using a general linear model in SPSS software 20.0 (IBM Corp., Armonk, NY, USA), and a 5% level of significant differences was determined using Duncan’s test. All experiments were replicated three times. The microbial diversity data were analyzed on the Illumina MiSeq PE300 platform/NovaSeq PE250 platform (Illumina, San Diego, CA, USA) and provided by Majorbio Bio-pharm Technology Co., Ltd. (Shanghai, China). Species taxonomy annotation was performed on OTUs, and the corresponding abundance information of each OTU annotation result was counted in each sample. Sobs index was a measure of estimating the microbial alpha diversity in the sample; a higher Sobs index value represents higher microbial diversity [30]. Principal component analysis (PCA) was used to assess microbial beta diversity, and classified at the genus level. The closer the distance between samples in the figure, the more similar the microbial compositions in the samples were. Information on the 50 most abundant species was clustered using OTU and a heatmap chart was created. RDA/CCA combined correspondence analysis with multiple regression analysis, and each step of the calculation was regressed with environmental factors (sensory quality scores and colony count) to reflect the relationship between the bacteria and environmental factors. RDA was based on a linear model and CCA was based on a unimodal model. Analysis can detect the relationship between environmental factors, samples, bacteria, or the relationship between the two.

## 3. Results

### 3.1. Sequencing Output

A total of 11 groups (33 samples) of mixed microorganisms in lettuce were collected from various stages of processing and storage. All samples met quality inspection standards. A total of 2,380,519 valid sequences were generated from a total of 33 bacterial DNA samples in lettuce and 1,887,633 high-quality sequences remained for analysis after screening and optimization. From the sequencing samples of the processing (fresh, washed, cut, then disinfected and dewatered) and storage (storage for 6 h, 12 h, 1 d, 2 d, 3 d, 4 d, 6 d and 8 d) groups, 514,809 and 1,372,824 high-quality sequences were obtained, respectively. The average quantity of high-quality sequences from all samples was 171,603 [(514,809 + 1,372,824)/11]. This indicated that the sequencing data was sufficient to reflect the microbial community contained in the sample. Sequence OTU clustering and notation were performed on the quality sequences at a 3% divergence level (Table 2). The taxonomic annotations of the species in the samples and the corresponding abundance information in each sample can be seen from the table. More than 400 OTUs were identified in lettuce samples using 16S rDNA sequencing, which is different from previous results of spinach studies. Researchers identified more OTUs (more than 600 OTUs) in spinach samples than in lettuce [31]. This indicated that there are differences in the abundance of microorganisms in different vegetables.

**Figure 1:**
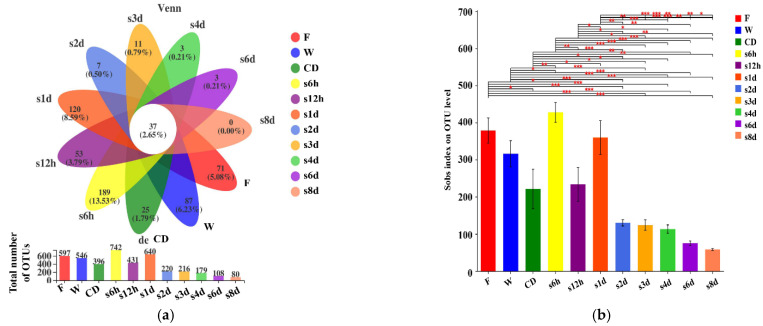
Venn diagram of OTU level among 11 groups (**a**). The petals are the number of species unique to the corresponding group, and the center is the number of species common to all groups. Sobs index of OTU level among 11 groups (**b**). The abscissa is the group name, and the ordinate is the exponential average of each group. The significant difference between the selected two groups of samples that with significant difference are marked, *p* ≤ 0.05 is marked as *, *p* ≤ 0.01 is marked as **, *p* ≤ 0.001 is marked as ***.

The OTU distribution of samples was shown in a Venn diagram (Figure 1a). There was a total of 606 types (71 + 0 + 3 + 3 + 11 + 7 + 120 + 53 + 189 + 25 + 87 + 37) of OTU in the 11 groups, with 37 OTUs shared among groups. In general, the total number of OTUs in the lettuce samples of fresh-cut and storage was lower than in fresh lettuce, and gradually decreased with the prolongation of storage time. This suggested that there were similarities in the microbial composition among samples, and processing and storage treatments can reduce the relative abundance of bacteria in the lettuce. The comparison of Sobs index for the 11 groups was shown in Figure 1b. The Sobs indices of the samples from storage groups were significantly lower than that of the raw material and the processing groups, except for the samples of pre-storage (6 h, 12 h and 1 d). This indicated a lower microbial diversity in the storage samples. This may be because the interstitial fluid overflow of lettuce, caused by the cutting treatment (disinfection and dewatering after cutting), as the hydrogen peroxide in the exudate causes temporary damage or inactivation of the microorganisms, and the disinfection process further reduced the number of cells [32]. After a period of storage, the passivated bacteria grow slowly again. The number of dominant bacteria increased, but the relative abundance of bacteria decreased [6,33,34]. After storage for 2 days, the number of microorganisms in the lettuce began to decrease, and gradually decreased with the extension of the storage period. This because some microorganisms are not suitable for low temperature growth [9].

### 3.2. Bacterial Compositions of Fresh-Cut Lettuce

**Figure 2:**
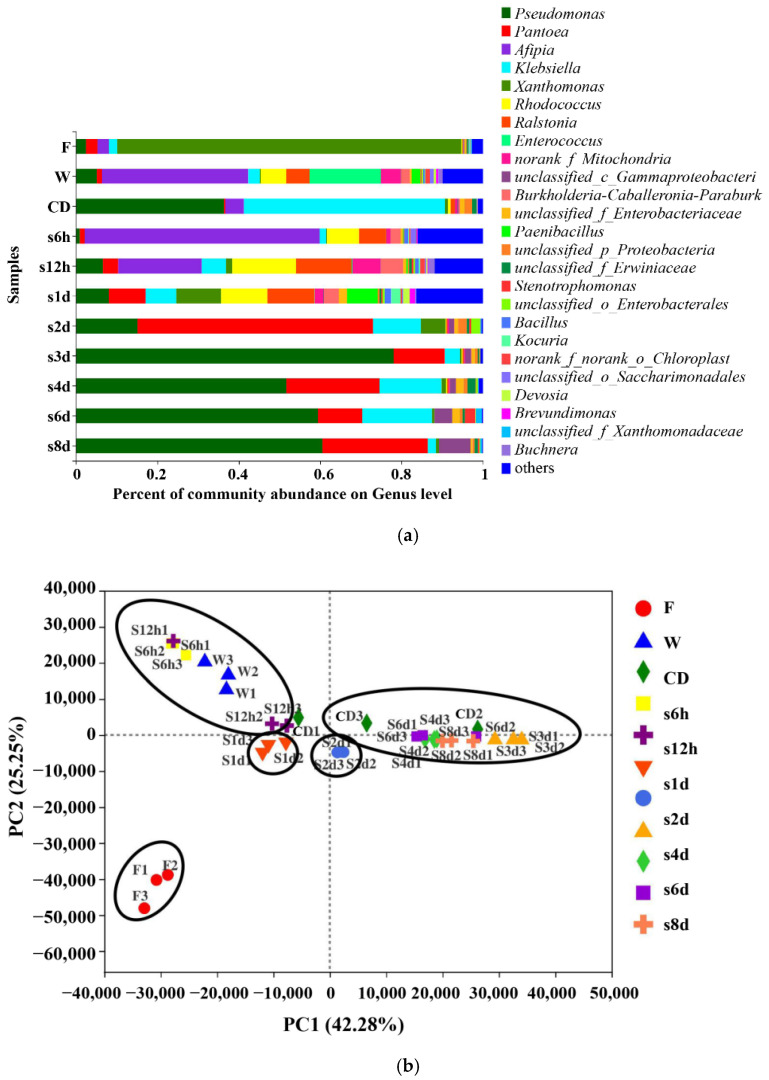
Community bar plot analysis of relative abundance percentage (%) of the bacterial genera in fresh-cut lettuce samples at each stage of processing and storage (**a**) and PCA analysis (**b**) on genus level.

The samples showed a high abundance at the genus level (Figure 2a). A total of six microorganism phyla and 21 microorganism species were found in 11 groups of samples, the microorganism phyla include: *Proteobacteria*, *Actinobacteriota*, *Firmicutes*, *Bacteroidota*, *Cyanobacteria*, and *Patescibacteria*. The dominant genus of fresh samples belonged to the *Xanthomonas* (84.55%) of the phylum *Bacteroides*. Studies have shown that *Xanthomonas* is a plant pathogen [35], and it could cause bacterial leaf spot of leafy vegetables, which would reduce the quality of lettuce and increase the possibility of loss after harvest, so to wash it away is beneficial [36]. Small amounts of *Klebsiella* and *unclassified_o_Enterobacterales* were also found in the lettuce samples, and this is consistent with the results analyzed in endive lettuce. The difference is that *Pseudomonas*, *Pantoea* and *Afipia* were also detected in the lettuce in this paper, while small amounts of *Serratia marcescens*, *Acinetobacter*, *Morganella* and *Serratia* were found in previous studies in endive lettuce [37]. For processing and pre-storage samples, the dominant genus belonged to the phylum *Proteobacteria*. The most dominant genus was *Afipia* (washing: 35.98%, s6h: 57.75%, s12h: 20.57%), *Klebsiella* (cutting then disinfection and dewatering: 49.44%) and *Ralstonia* (s1d: 11.61%), respectively. Oie et al. also detected the growth of *Pseudomonas* fluorescens and *Pseudomonas* aeruginosa on the vegetables [38]. Previous studies have shown that *Pseudomonas* is the main bacteria in the processing of endive lettuce. This is consistent with the finding in this research that *Pseudomonas* is the dominant bacteria in lettuce after dewatering [39]. Different from previous studies, the washing, cutting then disinfection and dewatering process in this paper reduced *Xanthomonas* in lettuce but did not remove *Pseudomonas*, and the relative abundance of *Pseudomonas* increased [40]. This may be because the washing treatment reduced the total number of colonies and more *Xanthomonas*, which increased the proportion of *Pseudomonas*. For post-storage samples, the OTU showed a low abundance. The dominant genus in most samples, except the 2 d sample (the dominant genus of 2 d samples was *Pantoea*), were similar, belonging to the *Pseudomonas* of the phylum *Proteobacteria*. However, the proportion of core genera showed a difference; the dominant genus changed from *Afipia* to *Pseudomonas* during storage. In general, the most abundant genus during processing and storage belongs to the phylum *Proteobacteria*, which is consistent with previous observation [41]. Researchers have shown that the relative abundance of bacteria changed along the processing chain and packaging storage time [42]. This study indicated that *Pseudomonas* was able to proliferate after acclimating to the environment in the later stage of storage. In addition to *Pseudomonas*, *Pantoea* and *Klebsiella* were also found in fresh-cut vegetables, which was consistent with previous findings from Efimochkina [43]. The difference was that the relative abundance of *Afipia* and *Ralstonia* was also detected to increase significantly after processing. They lettuce might have been contaminated by washing with water, which researchers reported as a potential source of *Afipia* and *Ralstonia* [44].

PCA of 33 samples in 11 groups is shown in Figure 2b. PCA results revealed that more variation was represented by the first principal coordinate axis (PC1) than the second axis (PC2) in samples. In terms of the differences in the distribution of PCA, the samples were distinctly grouped and the difference accounts for 67.73% of the total variation. The first principal component divided all samples into two groups, the fresh, washed and pre-storage (6 h, 12 h, 1 d) samples were divided into one group, and the cut, disinfected, and dewatered, and post-storage samples were divided into another group, indicating that they are relatively close to each other within the group. This result was consistent with the similarity of relative abundance among the samples in Figure 1a. The dominant genus in the washed lettuce was consistent with the samples stored for 6 h and 12 h, and the dominant genus after storage for 3d was consistent with the samples stored for 4 d, 6 d and 8 d. 

**Figure 3:**
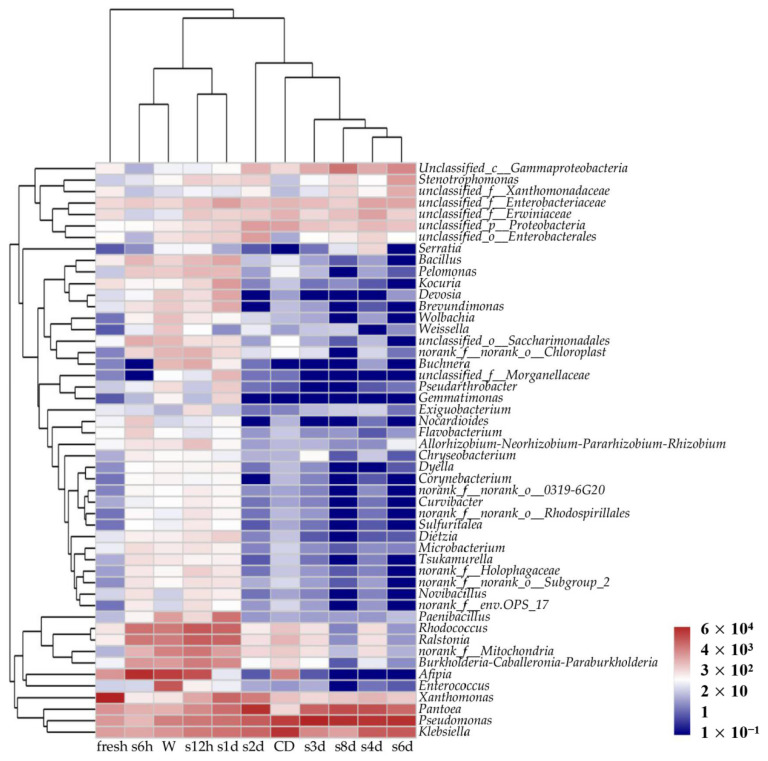
Heatmap chart and clustering results of 50 most abundant bacterial genera in different samples.

Clustering results showed that the overall distribution of the dominant genera could be easily divided into group 1 (fresh), group 2 (washed and stored for 6 h, 12 h and 1 d) and group 3 (cut, then disinfected and dewatered, and stored for 2 d, 3 d, 4 d, 6 d and 8 d) (Figure 3). The samples from group 2 were relatively close in the cluster, and the same was true for the samples from group 3. The relative abundance of *Burkholderia-Caballeronia-Paraburkholderia*, *Rhodococcus*, *Ralstonia*, *Bacillus* and *Pelomonas* of group 2 were higher than for fresh lettuce. The relative abundance of most species (including *Bacillus*, *Kocuria*, *Devosia*, *Brevundimonas*, *Pseudarthrobacter*, *Gemmatimonas*, *Exiguobacterium*, *Dietzia*, *Nocardioides*, *Flavobacterium*, *Allorhizobium-Neorhizobium*, *Enterococcus*, *Xanthomonas* and *Novibacillus*) of group 3 decreased compare to fresh lettuce, but the relative abundance of *Pseudomonas* was higher than group 1 and group 2. This might be because the low-temperature cold storage environment prevented the survival of some bacteria.

### 3.3. Enumeration of Microorganisms from Lettuce

The total number of colonies in fresh samples exceeded 5 log CFU/g, reduced to approximately 4 log CFU/g after washing, cutting then disinfecting and dewatering (Table 3). At the beginning of storage, the total number of colonies at 6 h and 12 h was the lowest (about 3 log CFU/g), then it gradually increased after storage for 1 d. The total number of colonies in samples stored for 3 d, 4 d, 6 d and 8 d exceeded 5 log CFU/g. In all samples, the number of *Bacillus cereus* was less than 100 CFU/g, *Staphylococcus aureus* was less than 3 MPN/g, the number of *E. coli* was less than 3 MPN/g and the number of *E. coli* O157:H7, *Salmonella* and *Listeria monocytogenes* was below the detection limit. This may be because these foodborne pathogens, which are considered to have a significant impact on the fresh produce industry, were so low that they could not be detected. It may also be attributed to the bactericidal effect of the sodium hypochlorite disinfectant reducing the number of pathogenic bacteria, which studies have shown are unlikely to be disinfectant-resistant microbiota [45]. Although the relative abundance of *Afipia*, *Burkholderia-Caballeronia-Paraburkholderia*, *Rhodococcus*, *Ralstonia*, *Bacillus* and *Pelomonas* were higher than in fresh lettuce, the total number of colonies in lettuce after processing decreased. This indicated that the processing treatment effectively reduced the total number of colonies in the lettuce. The total number of colonies decreased further during storage, which might have been caused by the low temperature inhibiting the growth of some bacteria. The total number of colonies gradually increased after storage for 2 d, which might be caused by the growth of *Pseudomonas*.

### 3.4. Sensory Quality Analysis

**Figure 4:**
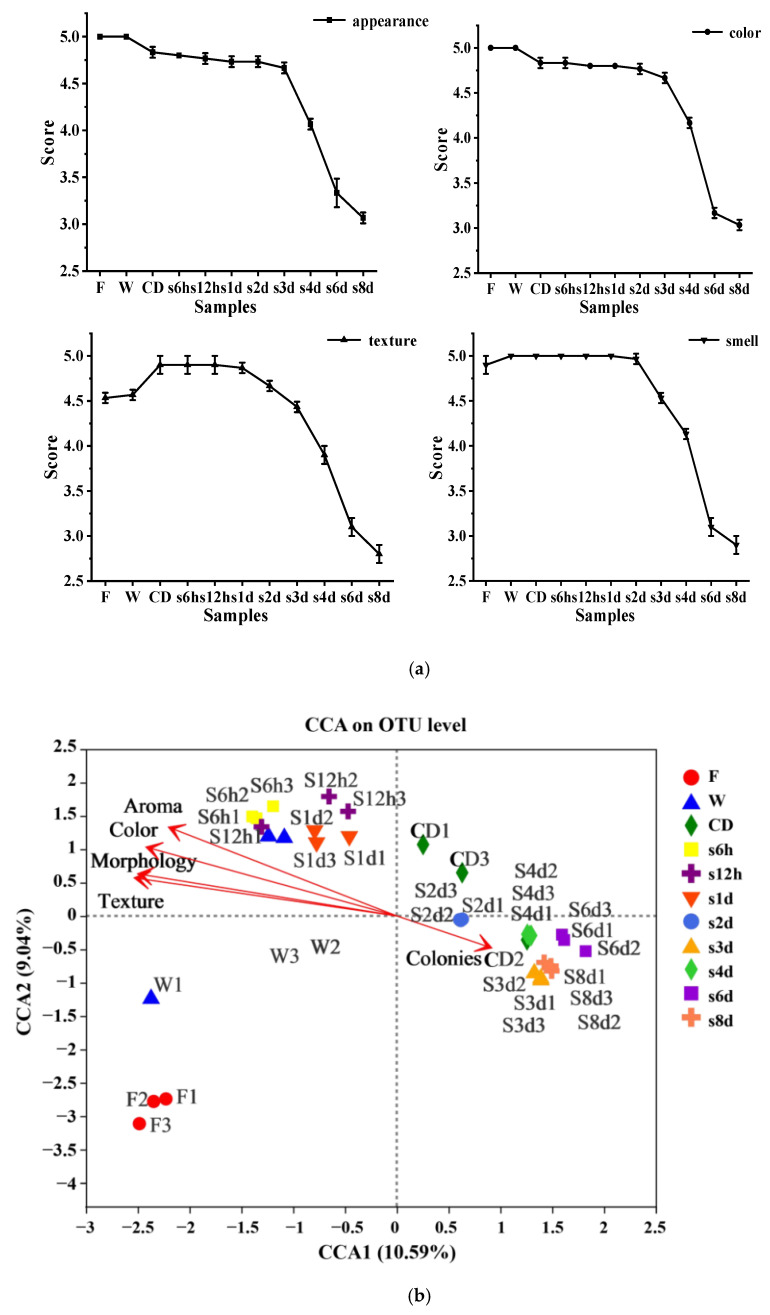
The average profile of each sensory index in different samples (**a**) and redundant analysis (RDA/CCA) of the relationship between the 50 most abundant bacterial microbiota and sensory quality of cucumbers in different samples (**b**). RDA is based on a linear model, and CCA is based on a unimodal model.

Fresh-cut lettuce was more likely to lose nutrients and water, causing a shrunken appearance, dimness of color, and smell deterioration during storage. Figure 4a showed the average profile of each sensory index (texture, morphology, aroma, color) of samples from different sampling points. The processed lettuce was the freshest, with the texture, morphology, aroma and color of fresh lettuce. After 3 d storage, the color and aroma of fresh-cut lettuce deteriorated further; the color was brown and there was a peculiar smell. After further storage, the sensory quality of lettuce continued to decline, the color was dim and the smell was unacceptable. The browning of lettuce may be the results of the enzymatic reaction in the vegetable. After cutting, the phenylalanine ammonia lyase (PAL) and peroxidase (POD) in the vegetable undergo dehydration and lignin synthesis, resulting in the color changes on the vegetable surface, off-flavor development and loss of firmness [46].

In order to determine the microbial diversity during processing and storage associated with sensory quality, a redundancy analysis (RDA) was performed for OTUs from the samples (Figure 4b). The total number of colonies and sensory quality were used as environmental variables, and the relative abundance of all OTUs in samples from different sampling points were used as species variables. Results showed that the sensory quality of the samples before storage for 1 day and processing was positively correlated with the predominant microbial bacteria, which might mean that the presence of *Afipia* and *Ralstonia* did not cause the sensory quality degradation of lettuce. However, there was a negative correlation between the sensory quality of samples after storage for 2 d and the predominant microbial bacteria, which might mean that the sensory quality of lettuce decreased with the presence of *Pseudomonas*. The higher the number of *Pseudomonas*, the worse the sensory quality of lettuce. This may be because the *Pseudomonas* is the most common psychrophilic species; they can produce enzymes that catalyze proteolysis and lipolysis reactions that contribute to the spoilage of refrigerated fresh produce, as well as pectolytic enzymes to degrade pectic substances of plant cell walls [41]. The presence of *Pseudomonas* promotes the degradation of the cell wall of lettuce and accelerates the degradation of the quality of lettuce [11]. Not only is it effective and important to take measures to control the total number of colonies, but the control of *Pseudomonas* is also more important.

## 4. Conclusions

The abundance and distribution of the bacteria changed during processing and storage. The abundance of bacteria in the processed and pre-storage (6 h, 12 h and 1 d) samples was high, and the relative abundance of bacteria in the post-storage samples was low. Washing could reduce the total colonies and *Xanthomonas* in the fresh lettuce, but it would also increase the risk of lettuce polluting microorganisms, *Afipia* and *Ralstonia*, from the water. With the extension of the low-temperature storage time, the total number of colonies increased and the dominant bacteria changed from *Afipia* to *Pseudomonas*. For the post-storage samples, the total number of colonies in the sample exceeded 5 log CFU/g, *Bacillus cereus* was less than 100 CFU/g, *Staphylococcus aureus* was less than 3 MPN/g, *E. coli* was less than 3 MPN/g, and *E. coli* O157:H7, *Salmonella* and *Listeria monocytogenes* were below the detection limit. Therefore, it is necessary to control and select the effective control method to reduce the number of total colonies and *Pseudomonas* in fresh-cut lettuce before storage for 1 d, and ultimately slow down the spoilage of fresh-cut lettuce. This study will provide guidance for the microbial control of fresh-cut lettuce.

## Figures and Tables

**Table 1:** Sensory evaluation criteria for samples.

Description	5 (Excellent)	4 (Good)	3 (Fair)	2 (Poor)	1 (Bad)
Appearance	Fresh	Dehydrated	Slightly water-soaked	Water-soaked	Rotten
Color	Fresh green	Green	Slightly dark	Dark	Dark yellow
Texture	Crisp	Slightly soft	Soft	Wilted on the edges	Wilted
Smell	Fresh	Slightly fresh	A little foul	A bit foul	Foul

**Table 2:** Results of sequence OTU clustering of quality sequences at a 3% divergence level.

Sample	OTUs	Phylum	Class	Order	Family	Genus	Species
F1	387	23	38	99	155	253	301
F2	408	20	38	94	145	253	317
F3	341	15	31	85	128	213	258
W1	281	18	33	79	123	189	227
W2	315	16	27	72	124	197	251
W3	352	21	40	90	145	227	282
CD1	241	20	31	76	118	173	199
CD2	262	17	30	76	119	180	214
CD3	161	15	24	57	82	115	135
s6h1	451	23	46	112	180	289	366
s6h2	399	24	43	110	167	259	327
s6h3	433	23	44	104	168	268	335
s12h1	181	17	23	65	94	127	146
s12h2	260	17	28	70	114	168	208
s12h3	260	18	32	80	123	182	216
s1d1	364	22	41	94	151	249	308
s1d2	312	16	28	71	117	196	245
s1d3	403	21	38	93	153	256	327
s2d1	135	12	19	40	69	93	106
s2d2	135	12	18	41	68	96	107
s2d3	120	10	14	39	61	82	93
s3d1	121	11	18	41	63	83	96
s3d2	111	12	18	41	64	78	87
s3d3	139	14	21	48	77	99	110
s4d1	103	9	12	30	51	70	80
s4d2	125	11	15	38	64	86	95
s4d3	111	8	13	37	56	76	87
s6d1	75	7	10	22	34	47	54
s6d2	69	8	11	24	36	45	49
s6d3	81	8	11	24	36	47	55
s8d1	55	5	7	17	26	34	38
s8d2	59	6	9	19	28	37	43
s8d3	60	7	9	19	29	37	42

The numbers 1, 2 and 3 after the sample name represent three replicate samples under the same treatment.

**Table 3:** The number of colonies in different samples.

Sample	Total Colonies (CFU/g)	*B. cereus* (CFU/g)	*S.aureus* (MPN/g)	*E. coli* (MPN/g)	*E. coli* O157: H7	*Salmonella*	*L. monocytogenes*
F	5.33 ± 0.20 bc	<100	<3	<3	ND	ND	ND
W	4.04 ± 0.04 def	<100	<3	<3	ND	ND	ND
CD	4.00 ± 0.38 def	<100	<3	<3	ND	ND	ND
s6h	3.26 ± 0.24 ef	<100	<3	<3	ND	ND	ND
s12h	2.97 ± 0.03 f	<100	<3	<3	ND	ND	ND
s1d	4.29 ± 0.89 cde	<100	<3	<3	ND	ND	ND
s2d	4.37 ± 1.30 cd	<100	<3	<3	ND	ND	ND
s3d	5.15 ± 0.46 bc	<100	<3	<3	ND	ND	ND
s4d	6.08 ± 0.21 ab	<100	<3	<3	ND	ND	ND
s6d	6.39 ± 0.48 a	<100	<3	<3	ND	ND	ND
s8d	7.05 ± 0.80 a	<100	<3	<3	ND	ND	ND

ND means not detected. Different letters represent the statistically significant differences between the different treatment groups *(p* < 0.05). *Bacillus cereus* is represented by *B. cereus*, *Staphylococcus aureus* is represented by *S. aureus*, *Escherichia coli* is represented by *E. coli*, *Escherichia coli* O157:H7 is represented by *E. coli* O157:H7, *Listeria monocytogenes* is represented by *L. monocytogenes*.

## Data Availability

The data presented in this study are available on request from the corresponding author.
